# Arabinogalactan-Proteins from the Liverwort *Marchantia polymorpha* L., a Member of a Basal Land Plant Lineage, Are Structurally Different to Those of Angiosperms

**DOI:** 10.3390/plants8110460

**Published:** 2019-10-29

**Authors:** Kathrin Happ, Birgit Classen

**Affiliations:** Pharmaceutical Institute, Department of Pharmaceutical Biology, Christian-Albrechts-University of Kiel, Gutenbergstr. 76, 24118 Kiel, Germany; khapp@pharmazie.uni-kiel.de

**Keywords:** AGP-antibodies, arabinogalactan-protein, bryophyte, cell wall, liverwort, *Marchantia polymorpha*, terrestrialization

## Abstract

The thalloid liverwort *Marchantia polymorpha* as a member of a basal land plant lineage has to cope with the challenge of terrestrial life. Obviously, the plant cell wall has been strongly involved in the outstanding evolutionary process of water-to-land-transition. AGPs are signaling glycoproteins of the cell wall, which seem to be ubiquitous in seed plants and might play a role in adaption to abiotic and biotic stress situations. Therefore, we investigated the cell wall composition of *Marchantia polymorpha* with special focus on structural characterization of arabinogalactan-proteins. The *Marchantia* AGP shows typical features known from seed plant AGPs like precipitation with β-glucosyl-Yariv’s reagent, a protein moiety with hydroxyproline and a carbohydrate part with 1,3,6-linked galactose and terminal arabinose residues. On the other hand, striking differences to AGPs of angiosperms are the occurrence of terminal 3-*O*-methyl-rhamnose and a highly branched galactan lacking appreciable amounts of 1,6-linked galactose. Binding of different AGP-antibodies (JIM13, KM1, LM2, LM6, LM14, LM26, and MAC207) to *Marchantia* AGP was investigated and confirmed structural differences between liverwort and angiosperm AGP, possibly due to deviating functions of these signaling molecules in the different taxonomic groups.

## 1. Introduction

Around 450 mya ago, land plants evolved from a freshwater alga of the charophyte lineage, and by the end of the Devonian (360 mya), the extant lineages of land plants (hornworts, liverworts, mosses, lycophytes, monilophytes, and spermatophytes) were established and today dominate the terrestrial environment [1,2,3,4]. The transition from water to land exposed plants to new conditions which needed key physiological and structural changes. The ancestors of extant bryophytes were among the earliest lineages of plants and therefore, studies on species of this lineage can offer insights into plant terrestrialization, one of the most foundational events in the history of life on earth. Although it is accepted that bryophytes (hornworts, liverworts, and mosses) are basal within the land plants, the relationship of hornworts, liverworts, and mosses to the monophyletic vascular plant group is still under discussion [3] with up to seven alternative hypotheses [5,6]. However, discovery of fossils suggest that the first land plants possessed features of liverworts [7,8,9], which makes *Marchantia polymorpha* an interesting model organism relating to plant evolution [10]. Genome sequencing of this liverwort species revealed insight into the origin of some fundamental plant properties, e.g., with regard to plant hormone signaling pathways of auxin, jasmonic acid, abscisic acid, and salicylic acid which are involved in regulation of key processes of land plants like growth, development, and biotic and abiotic stress [2,11]. It is reasonable to assume that the conquest of land required severe changes in cell wall composition [12]. Based on transcriptome data for cell wall biosynthesis glycosyltransferases, there is genetic evidence that many important cell wall polysaccharides already existed in the charophytes, which supports the hypothesis that charophytes were pre-adapted to life on land [13]. On the other hand, knowledge on the cell wall composition of liverworts is still limited [14]. Besides polysaccharides, arabinogalactan-proteins (AGPs) are important components of plant cell walls with functions in growth, development, reproduction, and also in adaption to abiotic and biotic stress [15,16,17,18]. They are highly glycosylated members of the family of hydroxyproline-rich glycoproteins (classified in [19]) and seem to be present in all seed land plants. Their carbohydrate moieties are composed mainly of arabino-3,6-galactans (type II AGs; normally around 90% of the molecule) which are covalently linked via hydroxyproline (Hyp) to relatively small protein/peptide backbones (normally around 10% of the molecule). A typical feature of AGPs is their ability to precipitate with Yariv phenylglycosides, e.g., the β-glucosyl Yariv reagent. Structural investigations on AGPs from spore land plants are limited, but they have been found in some species of monilophytes, lycophytes, and bryophytes [20]. One typical feature of moss and fern AGPs, the occurrence of the unusual monosaccharide acofriose (3-*O*-Me-Rha) has been lost during evolution and is absent in angiosperm AGPs. With regard to bryophytes, only AGPs from some moss species have been isolated and analytically characterized. Although AGPs have been detected in one species of hornworts [21] and different species of liverworts [20] by Yariv’s reagent or monoclonal antibodies directed against glycan motifs present in arabinogalactans, no AGP has been isolated and analyzed from a horn- or a liverwort up to now. Therefore we characterized the general composition of the cell wall of the early land plant *Marchantia polymorpha* with special focus on AGPs. The results broaden the knowledge on plant cell wall evolution, especially with regard to the conquest of land.

## 2. Material and Methods

### 2.1. Plant Material

The liverwort *M. polymorpha* (Carl von Linné) was collected in the Botanical Garden of the Pharmaceutical Institute in Kiel and the Botanical Garden of the Christian-Albrechts-University of Kiel in June 2018. The collected material of *M. polymorpha* contained the whole plant, including thalli, rhizoids, and male/female gametophores. It was cleaned with water and freeze-dried.

### 2.2. Isolation of Different Fractions of the Cell Wall

The freeze-dried *M. polymorpha* material was milled and freed from polyphenols with two consecutive aceton extractions. 70% Aceton was added to the plant material in the ratio 1:10 (w/v), incubated for 21 h at 4 °C under stirring and removed by vacuum filtration. The following aqueous extraction in the ratio 1:10 (w/v) of the dried plant material carried out for 24 h at 4 °C and continuous stirring. After removing the insoluble residue from the aqueous extract with a tincture press, the insoluble residue was extracted with 0.2 M ammonium oxalate ((NH_4_)_2_C_2_O_4_), followed by 3% (w/v) sodium carbonate (Na_2_CO_3_) and with 2 M potassium hydroxide (KOH) [22,23]. Each extraction was done at 70 °C under stirring for 21 h and was centrifuged at 3000 g for 10 min.

The extracted fraction were proceed in different ways. The aqueous extract was used for the isolation of the high molecular weight fraction (HMF). Therefore, the aqueous extract was heated at 90–95 °C for 10 min to denatured proteins (AGPs stay soluble). The denatured proteins were removed by centrifugation at 4122 g for 20 min. The aqueous, protein free extract was poured into 4 °C cold absolute ethanol up to a concentration of 80% (v/v) ethanol in order to precipitate the HMF including polysaccharides and AGPs. The precipitation was isolated by centrifugation at 4122 g, 4 °C for 30 min and freeze-dried. Isolation of the AGP from HMF was achieved by selective precipitation with β-glucosyl-Yariv reagent (βGlcY) (see below).

The (NH_4_)_2_C_2_O_4_ extract was evaporated under reduced pressure to 100–200 mL and was dialyzed. The Na_2_CO_3_ extract was used for a precipitation with acetone (80% (v/v)), whereby the precipitate was resuspended in deionized water and dialyzed. The KOH extract was neutralized with 37% hydrochloric acid and was evaporated under reduced pressure to 100–200 mL prior to dialysis. All extracts were dialyzed (Visking^®^ dialysis tubing, 7 kDa MWCO, Medicell Membranes Ltd., London, UK) against changes of deionized water in 4 °C for a total of 4 days, freeze-dried, and weighed. The extracts were stored at −28 °C.

### 2.3. Isolation of AGP

The isolation of AGP with βGlcY was performed according to [24]. HMF was dissolved in distilled water and precipitated by adding an equal volume of an aqueous solution of 1 mg/mL βGlcY and 0.3 M NaCl. After precipitation overnight at 4 °C the AGP-Yariv-complex was isolated by centrifugation (19000× *g*, 30 min, 4 °C) and dissolved in distilled water. To decompose the AGP-Yariv-complex sodium hydrosulfite was added at 50 °C until the red color disappeared. After cooling the AGP fraction was dialyzed against deionized water at 4 °C (Visking^®^ dialysis tubing, 14 kDa MWCO, Medicell Membranes Ltd., London, UK) and the resulting AGP fraction and the residual HMF was freeze-dried.

### 2.4. Gel Diffusion Assay

For cavities were stamped in an agarose gel (10 mM Tris-HCl, 1 mM CaCl_2_, 0.9% NaCl, 1% agarose) and filled with the red-colored βGlcY (middle cavity, 1 mg mL^−1^), surrounded by AGP from *Echinacea purpurea* (10 mg mL^−1^), βGlcY precipitated fraction from *Marchantia polymorpha* (10 mg mL^−1^), and the supernatant of the βGlcY precipitation of *Marchantia polymorpha* (100 mg mL^−1^). A red precipitation line indicates the presence of AGP.

### 2.5. Hydrolysis of AGP with Trifluoroacetic Acid (TFA)

Samples were dissolved in 0.5 M TFA in concentration of 10 mg mL^−1^. The partial hydrolysis was carried out for 2 h at 80 °C in Wheaton vials on the heating block (Wheaton^®^ V-Vial; Bioblock Scientific, Thermolyne Corp., Thermo Scientific, Waltham, MA, USA). To stop the reaction distillated water was added. Then water and TFA were removed by vacuum evaporation. This was followed by precipitation in 80% EtOH. The sample evaporated to dryness was taken up in distillated water and added to the 4-fold amount of 99% ethanol. After precipitation over night at 4 °C the precipitate was isolated by centrifugation (19,000× *g*, 4 °C, 20 min), was dissolved in distillated water and was freeze-dried.

### 2.6. Determination of Uronic Acids

The content of uronic acids was determined photometrically according to [25].

### 2.7. Analysis of Neutral Monosaccharides

Qualitative and quantitative monosaccharide composition was determined according to [26]. 2–3 mg sample with 0.5 mg of internal standard *myo-*inositol were hydrolyzed with 2 M TFA, reduced with sodium borhydride and acetylated with 1-methylimidazole and acetic anhydride. Identification and quantification of monosaccharides was performed by gas liquid chromatography (GLC) with flame ionization detection (FID) and mass spectrometry (MS) (GC + FID: Agilent 7890B, Agilent Technologies, USA; MS: Agilent 5977B MSD, Agilent Technologies, USA; column: Optima-225, 25 m, flow rate: 1 mL min^−1^; temperature 230 °C; split ratio 30:1). Peak identification was done in comparison to standard monosaccharides (relative retention times to inositol), 3-O-Rha was identified by MS. For quantification, response factors determined by a standard monosaccharide mixture were used.

### 2.8. Structure Elucidation of Arabinogalactan Moiety

Structure elucidation of AGP and the product after partial acid hydrolysis was performed by methylation analysis according to [27]. The partially methylated alditol acetates (PMAAs) were separated via GC (instrument: Agilent 7890B, Agilent Technologies, USA; column: Optima-1701-0.25 µm, Machery & Nagel, Düren, Germany; flow rate: 1 mL min^−1^; temperature: initial 170 °C for 2 min, with rate 1 °C min^−1^ to 210 °C, then with rate 30 °C min^−1^ until 250 °C is reached and following hold time of 10 min) and analyzed by MS and FID. Identifications were based on peak retention times and on comparison of mass spectra with the spectra from a library of undermethylated reference compounds, established in our working group. The quantification of the PMAAs was done by integration of the corresponding FID-signal areas. Mass percentage was converted into molar percentage by using molar response factors for FID [28].

### 2.9. Elemental Analysis

Quantitative determination of nitrogen in Yariv fraction was performed with HEKAtech CHNS Analyzer (Co. HEKAtech, Wegberg, Germany). Before analysis aminobenzenesulfonamide was used to calibrate the system. 2 mg Yariv fraction were burned in an excess of oxygen and combustion products were analyzed, while a blank value was measured under same conditions with an empty tin capsule.

### 2.10. Determination of Hyp Content

Colorimetric quantification of Hyp was done according to [29]. After acid hydrolysis (6 M hydrochloric acid, 110 °C, 22 h in a Wheaton^®^ V-Vial), the sample was oxidized by chloramine-T, coupled to p-dimethylaminobenzaldehyde in strong perchloric acid and the absorption of the colored product measured at 558 nm (UVmini-1240, Shimadzu AG, Kyoto, Japan). A calibration line was established 4-hydroxy-L-proline.

### 2.11. Determination of Molecular Weight

The absolute molecular mass of AGP was determined with SEC-MALS. For the analysis an Äkta pure chromatography system (GE Healthcare Bio-Sciences, Marlborough, MA, USA) was used with Superose 6 Increase 10/300 GL column (GE Healthcare Bio-Sciences, USA), a multi-angle light scattering detector (MALS, DAWN8+, Wyatt Technology Corporation, Santa Barbara, CA, USA) and a refractive index detector (Optilab T-rEX, Wyatt Technology Corporation, USA). The column was equilibrated with elution buffer (0.15 M NaCl, 0.05 M phosphate buffer, pH 7.0) at a flow rate of 0.5 mL min^−1^. Yariv fraction was dissolved in elution buffer (2 mg mL^−1^) and incubated overnight. 100 µL samples were injected and eluted with elution buffer.

### 2.12. Enzyme-Linked Immunosorbent Assay (ELISA)

The affinity of different antibodies was tested in an indirect ELISA. Therefore 96-well plates (Nunc^®^, Nalge Nunc International, Roskilde, Denmark) were coated with AGP or TFA hydrolyzed AGP (100 µL per well) in the concentration 0, 2.5, 5, 10, 25 µg mL^−1^ at 37.5 °C for 3 days. The plates were washed three times with PBS-T (pH 7.4, 0.05% Tween^®^ 20) and blocked with 1 w/v % () BSA in PBS (pH 7.4, 200 µL per well, 1 h at 37.5 °C). After three washing steps 100 µL of the primary antibody in 1:5 dilution (KM1) [30] or 1:20 dilution (JIM13, LM2, LM6, LM14, LM26, MAC207) were added and incubated for 1 h at 37.5 °C. The plates were washed again three times wih PBS-T. The secondary antibody (Anti-Mouse-IgG or Anti-Rat-IgG conjugated with alkaline phosphatase, Sigma-Aldrich Chemie GmbH, Taufkirchen, Germany) was incubated 1 h at 37.5 °C in a dilution of 1:500 in PBS. After incubating and washing, 100 µL of the substrate p-nitro-phenylphosphate (Alkaline Phosphatase Yellow (pNPP) Liquid Substrate System, Sigma-Aldrich Chemie GmbH, Taufkirchen, Germany) were added in each well. Absorption was measured at 405 nm in an ELISA reader (Tecan Spectra Thermo, Männedorf, CH). Absorptions of negative control (without AGP) were subtracted. Samples were tested in triplicate.

## 3. Results and Discussion

### 3.1. Isolation of Different Fractions of the Cell Wall

Cell walls were sequentially extracted following extraction protocols described previously [22,23] with a variation of solvents (see also Section 2.2). After extraction of water-soluble polysaccharides, pectic fractions are solubilized in hot oxalate and also in sodium carbonate, whereas potassium hydroxide is used to extract hemicelluloses. Yields of the different polysaccharide classes (m m^−1^ related to dry plant material) were 6.7% for the water-soluble fraction, 3.1% for the oxalate extract, 3.8% for the sodium carbonate and 17.5% for the KOH extracted fractions. The neutral monosaccharide composition and the uronic acid content of the different fractions are shown in Table 1 and Table 2.

Dominant monosaccharides in the water soluble fraction were Glc, Gal, and Ara, possibly due to presence of glucans and arabinogalactan(-protein)s. The oxalate and carbonate fractions reflect the composition of pectic polysaccharides with uronic acids and the neutral monosaccharides Gal, Ara, and Rha, although the amounts of uronic acids are rather low compared to pectins from seed plants. Furthermore, there were appreciable amounts of Glc in these fractions. In the oxalate fraction, a small peak appeared at the retention time of ribose. Finally, the KOH extraction yielded a fraction probably composed of different hemicellulosic polysaccharides like mannans and xyloglucans. All fractions contained small amounts of 3-*O*-Me-Rha, which was identified by the retention time of its alditol derivative and the corresponding mass spectrum (see below Section 3.2.3).

Cellulose, xylans, mannans, xyloglucans, pectic polysaccharides, and AGPs have been shown to be present in bryophyte cell walls [31,32,33,34,35,36,37]. On the other hand, there are differences to seed plants, e.g., cell walls of bryophytes cannot be clearly distinguished as primary or secondary walls [14], bryophyte walls contain less than 1% of the amount of RG-II that is present in the walls of tracheophytes [34,38], and no lignin is detectable in these walls, although all putative lignin biosynthesis genes except ferulate-5-hydroxylase have been identified [2]. Furthermore, the unusual monosaccharide acofriose (3-*O*-Me-Rha) not present in angiosperms occurs in cell walls of bryophytes [31,35,39,40]. For *Marchantia*, knowledge on cell wall composition is very limited. Konno et al. [41] isolated different pectic fractions from *M. polymorpha* cell cultures with compositions comparable to our results and proposed the presence of homogalacturonan, rhamnogalacturonan, and a glucose-rich polymer. Identification of pectin methyl transferases in the genome of *M. polymorpha* is another proof of presence of pectins (Bowman et al., 2017). Xyloglucans from *M. polymorpha* have been detected using a monoclonal antibody [42] and have been carefully characterized [43]. Interestingly, the liverwort *M. polymorpha* and also the moss *Physcomitrella patens* possess XXGGG- and XXGG-type xyloglucans with side chains that contain GalA, whereas hornworts synthesize XXXG-type xyloglucans comparable to vascular plants. This is in accordance with the presence of appreciable amounts of uronic acids in the hemicellulosic fraction we isolated from *Marchantia* cell wall. Genomic data revealed presence of enzymes involved in xyloglucan biosynthesis [2].

### 3.2. Isolation and Characterization of AGPs

#### 3.2.1. Yield and Gel Diffusion Assay

From the HMF, we isolated an AGP fraction by precipitation with Yariv’s reagent which accounted for 0.18% of the dry mass of the liverwort. To the best of our knowledge, this is the first time an AGP has been isolated and characterized from a liverwort. The yield is comparable to AGP content in the mosses *Sphagnum* and *Physcomitrella*, but three-fold higher compared to *Polytrichastrum* [31].

**Figure 1:**
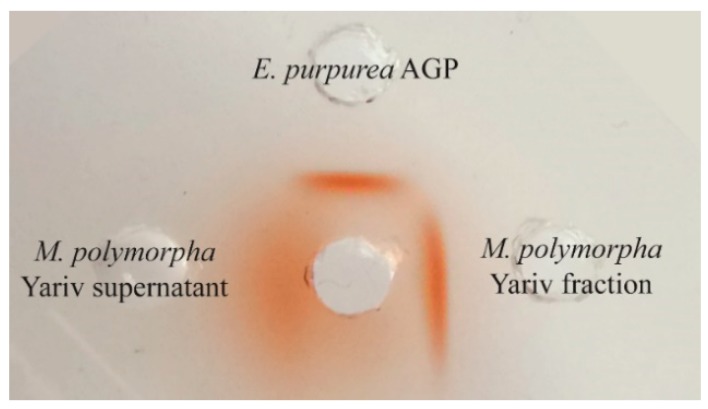
Gel diffusion assay with Yariv-fraction (10 mg/mL) and Yariv-supernatant (100 mg/mL) of *M. polymorpha* compared to *Echinacea purpurea* AGP (10 mg/mL).

The gel diffusion assay with Yariv´s reagent showed a strong band for the Yariv-precipitated fraction of *M. polymorpha* even a little bit stronger compared to AGP from *Echinacea purpurea* and no clear precipitation line for the Yariv-supernatant, which shows that no or only small amounts of AGPs remained in this fraction (Figure 1).

#### 3.2.2. Analyses of the Protein Moiety

Elemental analysis revealed an amount of nitrogen in the AGP of 3.99% which corresponds to a protein content of 24.9% according to Kjeldahl (× 6.25). This is very high compared to seed plant AGPs were in general the protein accounts for around 10% of the molecule [15]. High levels of protein have also been detected in AGPs of the moss *Polytrichastrum formosum* (18%) and the lycophyte *Lycopodium annotinum* (17%). Bioinformatic searches for AGP protein backbones already revealed the presence of some of these proteins in liverworts [44,45].

The amino acid hydroxyproline, which is responsible in AGPs for *O*-glycosidic linkage of the AG moieties to the protein, was quantified photometrically [29] and found to be 0.31% of the AGP, which means that Hyp accounts for only 1.25% of the protein moiety. Although this is rather low compared to seed plant AGPs, where the amount of Hyp is often up to 10% of the protein, there is enough Hyp residue for binding of the AG moieties in *Marchantia* AGP. As there is great variety in amino acid composition of AGP protein backbones, the amount of Hyp in AGPs is not strictly related to the amount of the AGP protein moiety.

#### 3.2.3. Analyses of the Carbohydrate Moiety

The carbohydrate moiety of the Yariv-precipitated fraction reflected the typical composition of an AGP with Gal and Ara as the main monosaccharides accounting for nearly 80% of the neutral monosaccharides (Table 3). Furthermore, Glc is present in appreciable amounts and also Rha, half of it with methylation at O-3 (= acofriose, see above). Other monosaccharides (Rib, Fuc, Xyl, and Man) are present in very small amounts and might be part of other polysaccharides not completely separated during centrifugation of the Yariv precipitate. The content of uronic acids was determined photometrically and found to be 4.4%. The supernatant of the Yariv precipitation still contained high amounts of Gal and Ara accompanied by nearly the same amount of Xyl and lower amounts of Glc, Fuc, Rha, and Man. For further structure elucidation, a partial hydrolysis of the Yariv fraction was performed (Table 3). Treatment with weak trifluoroacetic acid led to nearly complete loss of arabinose and slight loss of rhamnose and methylrhamnose, whereas the residual monosaccharide composition remained nearly unchanged.

**Figure 2:**
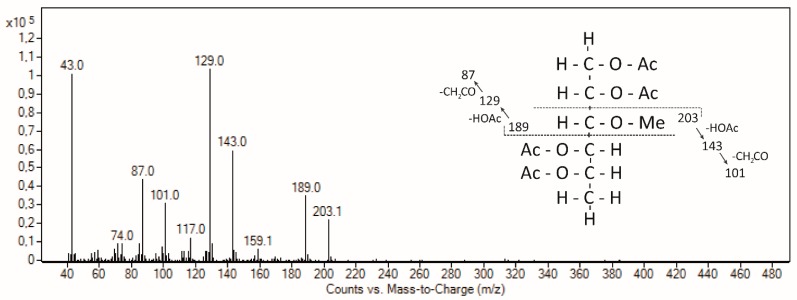
Mass spectrum and fragmentation pattern of 3-*O*-Me-Rha in the Yariv fraction of *M. polymorpha.*

In accordance with AGPs from different moss genera [31,39], acofriose is also part of *Marchantia* AGP (Table 3) and was clearly identified by retention time and mass spectrum (Figure 2). Compared to three AGPs from mosses covering the genera *Sphagnum*, *Physcomitrella*, and *Polytrichastrum*, which hold different positions in the phylogenetic tree of mosses (early, intermediate, and late diverging, respectively), the monosaccharide composition shows no general evolutionary trend; e.g., 3-*O*-Me-Rha is low in *Marchantia* and *Polytrichastrum* and five-fold higher in *Sphagnum* and *Physcomitrella*, whereas the content of Ara is high in *Marchantia* and *Physcomitrella* and two- to three-fold lower in *Polytrichastrum* and *Sphagnum* (Table 4). Fu et al. [39] speculated that this relatively nonpolar monosaccharide which is located at the outer surface of the molecule is likely to have some effect on the overall polarity of an AGP and that 3-*O*-Me-Rha residues in *Physcomitrella* AGPs might enable a function involving hydrophobic interactions which might be essential in mosses but not in angiosperms.

In contrast to broad knowledge on AGPs in seed plants, insight in occurrence and structure of AGPs in spore producing land plants (bryophytes, lycophytes, and monilophytes) is still very limited [20]. A plant transcriptome project revealed presence of sequences of GPI-anchored AGPs in liverworts, mosses, hornworts, lycophytes, and monilophytes [44], and a bioinformatic search for fasciclin-like arabinogalactan-protein backbones identified 14 such genes in *Marchantia polymorpha* [45]. For some liverwort species [21,46,47] and especially *Marchantia polymorpha* [48,49], presence of AGPs has been proven by detection with either Yariv´s reagent or with monoclonal antibodies directed against glycan motifs present in AGPs. Isolation and structural investigations on AGPs from bryophytes are restricted to some mosses [21,31,39,50].

#### 3.2.4. Structure of AGP

AGP of *M. polymorpha* was investigated for linkage types by methylation before and after partial acid hydrolysis (Table 5). The carbohydrate moiety of *Marchantia* AGP revealed the typical general composition comparable to an angiosperm AGP with the main components 1,3,6-linked Gal*p*, 1,3-linked Gal*p*, and terminal Ara*f*. Interestingly, the typical angiosperm AG-linkage type 1,6-linked Gal*p* is present only in traces, which indicates a highly branched structure. The unusual branching point 1,2,3-linked galactose, which is present in AGPs from the mosses *Polytrichastrum* and *Sphagnum* [31], has not been detected in *Marchantia* AGP. Furthermore, 1,4-linked Gal*p*, which has also been detected in moss and fern AGPs [31,39,51], is also present. After partial acid hydrolysis, there was complete loss of furanosidic arabinose and pyranosidic Rha which are mainly terminal and located in sidechains of the molecule. Furthermore, TFA acid hydrolysis led to strong increase of 1,6-linked Gal, proving that Ara is bound to Gal at C-3 of 1,3,6-linked Gal. Comparable to moss AGPs from *Physcomitrella*, *Polytrichastrum*, and *Sphagnum*, Rha*p* (including 3-*O*-Me-Rha*p*) is localized terminally [31,39], but also present in 1,4- and 1,2,4-linkage.

#### 3.2.5. Determination of Molecular Weight by Size-Exclusion Chromatography

Absolute molecular weight of *Marchantia* AGP was determined by gel permeation chromatography with RI- and MALS detection. For the main fraction with an absolute mass recovery of 49.4%, a molecular weight of 186 kDa was determined, followed by a peak with a mass recovery of 23.8% with a molecular weight of 84 kDa. In *Physcomitrella patens*, fractionation of the water-soluble AGP-fraction by anion-exchange chromatography yielded two AGPs with molecular weights of 224 and 100 kDa, respectively [39]. Molecular weights of the main fractions of *Sphagnum* and *Polytrichastrum* AGPs were 238 and 218 kDa, respectively, and accompanied by aggregates with molecular weights of over 1000 kDa [31].

### 3.3. Reactivity of *M. polymorpha* AGP with Antibodies Directed against Angiosperm AGPs in ELISA

**Figure 3:**
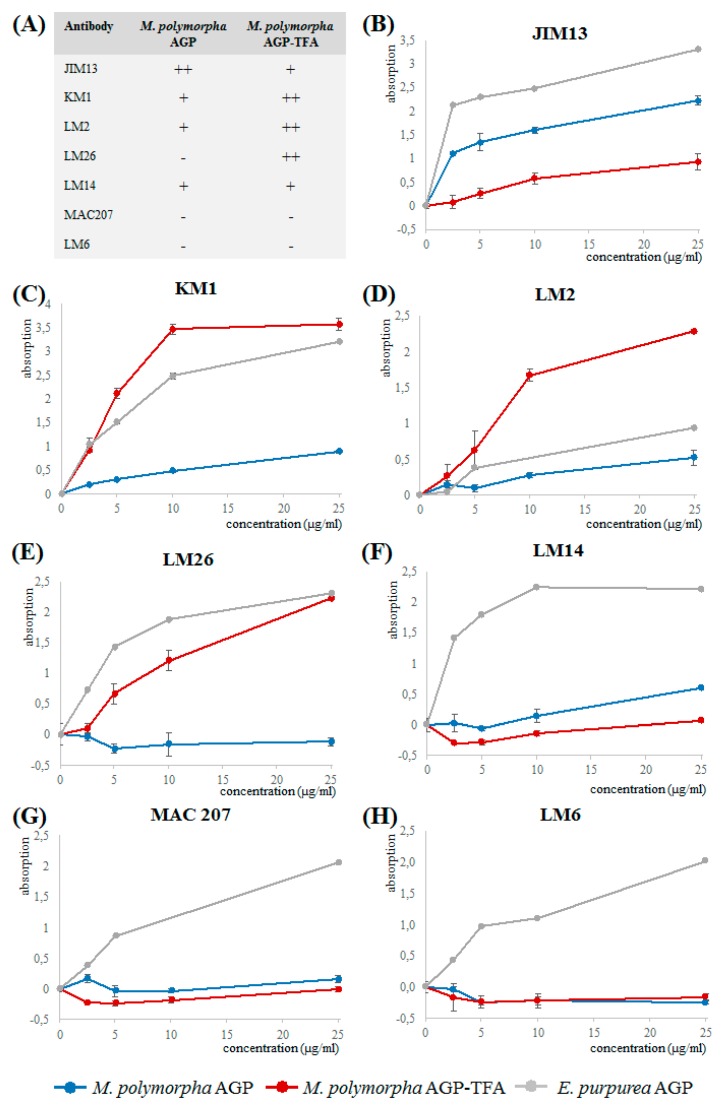
Reactivity of *Marchantia* AGP, partially degraded *Marchantia* AGP (AGP-TFA) and *Echinacea* AGP with antibodies directed against AG glycan motifs in ELISA. (**A**) Overview on reactivity of *Marchantia* AGP and *Marchantia* AGP-TFA with the different antibodies. (**B**) JIM13. (**C**) KM1. (**D**) LM2. (**E**) LM26. (**F**) LM14. (**G**) MAC207. (**H**) LM6.

Binding of the native *Marchantia* AGP and its partially degraded product to the antibodies LM2, LM6, LM14, LM26, MAC207, JIM13, and KM1 (epitopes and references see Table 6) was investigated in ELISA (Figure 3). Whereas JIM 13, MAC207, KM1, LM2, and LM14 are antibodies directed against AG glycan motifs present in angiosperm AGPs, LM6 recognizes 1,5-linked arabinose (present in arabinans but also in some AGPs) and LM26 is directed against a 1,4-linked galactan with branching at position C6.

Whereas *Echinacea* AGP reacted with all tested antibodies, the native *Marchantia* AGP shows no (LM6, MAC207) or only weak binding to LM2, LM14, LM26, and KM1, thus underlining structural differences to seed plant AGPs. The lack of interaction with LM6 underlines our structural finding that in contrast to *Echinacea* AGP, 1,5-linked Ara is present in *Marchantia* AGP only in very small amounts (Table 4). Only JIM13 strongly binds to the native *Marchantia* AGP, which indicates that the epitopes of MAC207 and JIM13 should be different, although both recognize an acidic trisaccharide isolated from hydrolysate of gum karaya (Table 5).

After cleavage of furanosidic Ara residues by mild acid hydrolysis, the residual branched galactan-protein showed binding to LM2, LM26, and KM1, probably because Ara is not substantial part of the epitopes of these antibodies. For LM2 and KM1, it has been shown that 1,6-linked Gal*p* is necessary for binding, for LM2 in combination with terminal GlcA*p* [56]. The native *Marchantia* AGP has only small amounts of 1,6-linked Gal, which strongly increases after partial acid hydrolysis to a level even higher compared to the original antigen (*Echinacea* AGP). After partial acid hydrolysis, there is also strong increase in 1,4-linked Gal probably responsible for good binding to LM26.

### 3.4. Functions of AGPs in Bryophytes

Functions of AGPs in seed plants are highly diverse and include processes like growth, cell proliferation, programmed cell death, pattern formation, and plant microbe interactions [17]. In the moss *Physcomitrella*, AGPs are required for apical cell extension of protonemata [50] and are also involved in processes with regards to water-balance [62,63]. Furthermore, antibodies against glycan motifs of AGPs labelled hyaline cells in *Sphagnum leaves* [21] and water-conducting cells of different mosses and liverworts (antibody CCRC-M7; [47]). Initial information on functions of AGPs in *M. polymorpha* has been gained by experiments in cell cultures, where AGPs have been blocked by addition of the β-glucosyl-Yariv´s reagent to the media. The results indicate a role of AGPs in protonemata differentiation [48], cell plate formation [49], and in cell wall regeneration of cultured protoplasts [64].

## 4. Conclusions

Today, understanding of cell wall evolution is incomplete due to limited knowledge of cell wall structure of non-flowering plants. *M. polymorpha*, as a member of a basal land plant lineage and important model organism, holds the key to a better understanding of the early evolution of land plants.

Therefore, we investigated cell wall composition of this liverwort with special focus on arabinogalactan-proteins. This is the first time an AGP from a liverwort has been structurally characterized. It could be shown, that general features known from seed plants are present already at this evolutionary stage. On the other hand, special attributes like a highly branched galactan and occurrence of the unusual monosaccharide 3-*O*-Me-Rha were detected in liverwort AGP, possibly connected to deviating functions. Further investigations on AGPs from extant lineages of spore-producing land plants (hornworts, liverworts, mosses, lycophytes, monilophytes) as well as from their algal ancestors are challenges for the future.

## Figures and Tables

**Table 1:** Neutral monosaccharide composition of the extracts from *M. polymorpha* in % (mol mol^−1^).

Neutral Monosaccharide	*M. polymorpha* HMF (n = 3)		*M. polymorpha* (NH_4_)_2_C_2_O_4_ (n = 3)		*M. polymorpha* Na_2_CO_3_ (n = 3)		*M. polymorpha* KOH (n = 3)	
3-*O*-Me-Rha	1.6	±0.3	1.1	±0.0	1.0	±0.1	trace	
Rha	4.8	±0.1	5.7	±1.3	11.5	±0.7	7.0	±0.1
Fuc	5.7	±0.0	5.3	±0.1	3.2	±0.3	2.4	±0.0
Rib (?)	trace		4.6	±0.0	trace		trace	
Ara	17.7	±0.1	16.0	±0.3	24.7	±0.8	17.3	±0.1
Xyl	11.2	±0.3	10.7	±0.1	4.9	±4.1	15.1	±0.4
Man	6.8	±0.2	7.8	±0.1	8.9	±0.4	10.6	±0.2
Gal	20.1	±0.5	17.5	±0.3	33.0	±1.7	24.1	±0.1
Glc	32.2	±0.3	31.3	±0.3	12.9	±0.6	23.3	±0.3

trace: value < 1%.

**Table 2:** Colorimetric determination of uronic acids in the extracts from *M. polymorpha* in %(m m^−1^).

*M. polymorpha*	Uronic Acids
HMF	4.2 ± 0.3
(NH_4_)_2_C_2_O_4_	11.4 ± 0.2
Na_2_CO_3_	4.9 ± 0.2
KOH	7.8 ± 0.0

**Table 3:** Neutral monosaccharide composition of the Yariv-precipitated fraction, the corresponding supernatant and the partially hydrolyzed Yariv-fraction from *M. polymorpha* in % (mol mol^−1^).

Neutral Monosaccharide	*M. polymorpha* Yariv Fraction (n = 3)		*M. polymorpha* Yariv Supernatant (n = 3)		*M. polymorpha* Yariv Fraction TFA Hydrolysis (n = 1)
Gal	46.9	± 0.3	23.4	± 0.1	79.6
Ara	31.6	± 0.5	22.7	± 0.1	2.1
Glc	9.5	± 0.7	9.4	± 0.2	10.3
3-*O*-Me-Rha	2.5	± 0.2	2.1	± 0.2	1.5
Rha	2.4	± 0.1	8.0	± 0.1	1.6
Rib (?)	2.1	± 0.1	trace		-
Fuc	1.8	± 0.1	8.9	± 0.1	trace
Xyl	1.6	± 0.1	17.8	± 0.2	2.7
Man	1.6	± 0.0	7.6	± 0.1	2.0

trace: value < 1%.

**Table 4:** Neutral monosaccharide composition of Yariv-fractions from different bryophytes.

	Liverwort	Mosses		
Neutral Monosaccharide % (*w*/*w*)	*Marchantia polymorpha*	*Sphagnum* sp. *	Physcomitrella patens *	Polytrichastrum formosum *
Gal	46.9	65.4	41.0	64.1
Ara	31.6	9.7	33.3	15.6
Rha	2.4	3.3	1.5	8.4
3-*O*-Me-Rha	2.5	11.9	11.9	2.6
others	16.6	9.7	12.3	9.3
Ara:Gal	1:1.5	1:6.7	1:1.2	1:4.1

* published in [31].

**Table 5:** Linkage type analysis of *M. polymorpha* AGP before and after partial acid hydrolysis (% mol mol^−1^).

Monosaccharide	Linkage Type	*M. polymorpha* AGP	*M. polymorpha* AGP after Partial Acid (TFA) Hydrolysis
Gal*p*	1,3,6-	27.1	20.6
	1,6-	-	25.0
	1,4-	4.3	17.4
	1,3-	19.2	14.3
	1-	-	22.7
Ara*f*	1,5-	2.7	-
	1,3-	4.3	-
	1-	36.3	-
Rha*p*	1,2,4-	1.5	-
	1,4-	2.4	-
	1-	2.2	-

**Table 6:** Antibodies tested for binding to *Marchantia* AGP.

Antibody	Epitope	Key References
JIM13	AGP glycan, e.g., β-D-GlcA*p*-(1→3)-α-D-GalA*p*-(1→2)-α-L-Rha	[52,53,54]
MAC 207	AGP glycan, e.g., β-D-GlcA*p*-(1→3)-α-D-GalA*p*-(1→2)-α-L-Rha	[53,54,55]
KM1	(1→6)-β-D-Gal*p* units in AGs type II	[30,56]
LM2	(1→6)-β-D-Gal*p* units with terminal ß-D-GlcA*p* in AGP	[53,56,57]
LM6	(1→5)-α-L-Ara*f* oligomers in arabinan or AGP	[50,56,58,59]
LM14	Type II AG in pectin or AGP glycan	[60]
LM26	Branched (1→4)-β-D-galactan	[61]
